# Supplementary material for: Propagated Circulating Tumor Cells Uncover the Potential Role of NFκB, EMT, and TGFβ Signaling Pathways and COP1 in Metastasis
Source: Cancers (Basel). 2023 Mar 17;15(6):1831. doi: 10.3390/cancers15061831 (PMC10046547; doi:10.3390/cancers15061831)
Supplement: Supplementary file 1 [file cancers-15-01831-s001.zip › cancers-2203752-supplementary.pdf]

SUPPLEMENTARY MATERIALS

Patient's clinical summary:

Patient #19 was a 56-year-old male diagnosed with stage IV non-small cell lung adenocarcinoma with confirmed metastases to the vertebral bone. He was initially treated with 30Gy radiation to the vertebral lesion and systemic first-line combination chemotherapy (carboplatin and pemetrexed) and immunotherapy (pembrolizumab) with eventual progression of metastatic bony disease requiring vertebral surgery and femur surgery and radiation. A liquid biopsy for CTC culture was collected before initiation of second-line systemic therapy with docetaxel and ramucirumab. The patient ultimately developed numerous progressive hepatic and osseous metastases after 3 cycles of therapy, endured multiple hospitalizations for cord compression and cholangitis, and died 9 months after the initial diagnosis

Patient #21 was a 69-year-old male diagnosed initially with stage II sigmoid colon adenocarcinoma treated with curative-intent surgical resection and adjuvant chemotherapy. After two years, he was diagnosed with stage IV disease with oligometastatic recurrence in the soft tissue between the bladder and rectum that was HER2-amplified, microsatellite stable, and RAS and BRAF wildtype. He progressed locally on 2 lines of systemic therapy, underwent oligometastectomy with partial cystectomy and en bloc colon resection, but eventually had systemic progression in the lungs. He failed to respond to third-line HER-2 directed therapy. Liquid biopsy was collected for CTC culture during fourth-line systemic treatment on which he ultimately had progression of disease.

Patient #22 was a female with an 18-year history of ulcerative colitis who was diagnosed at age 31 with stage IV sigmoid colon cancer characterized as RAS and BRAF wildtype, HER-2 non-amplified, and microsatellite stable with metastases to the liver and pelvis. A liquid biopsy was collected for this study prior to initiation of first-line systemic treatment with FOLFOX and panitimumab. She ultimately underwent surgical resection of pelvic disease and a total abdominal colectomy after disease response to systemic therapy but ongoing ulcerative colitis symptoms and subsequently achieved stable disease status on maintenance systemic therapy for 12 months. Eventually, she had progression of disease in the liver, abdominal lymph nodes, peritoneum, and right atrium treated with various local surgeries and systemic therapy. She died after progression on fifth-line systemic therapy about 2 years after initial cancer diagnosis.

Patient #26 was a female diagnosed at age 82 with stage IV moderately-differentiated pancreas adenocarcinoma with metastases to the liver, lymph nodes, and lung. A liquid biopsy was collected for CTC culture prior to initiation of first-line, palliative, systemic treatment with gemcitabine and nab-paclitaxel. She only received one dose of systemic therapy after which her course was complicated by transaminitis and hyperbilirubinemia due to progressive liver disease resulting in biliary obstruction that was unamenable to local intervention. She was recommended best supportive care, pursued home hospice, and ultimately died 3 months after her initial diagnosis.

SUPPLEMENTARY FIGURES/TABLES

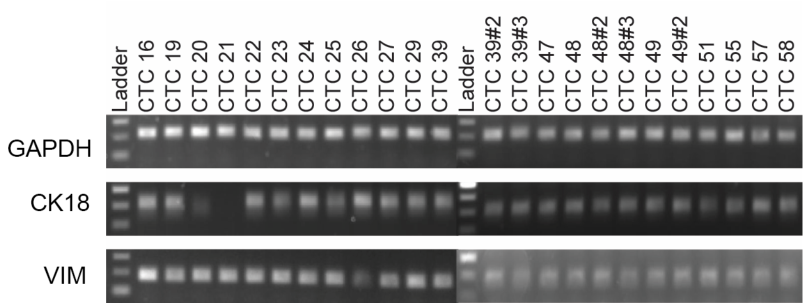

Figure S1. qRT-PCR was performed to confirm expression of human-specific cytokeratin 18 and vimentin in various cultured CTCs.

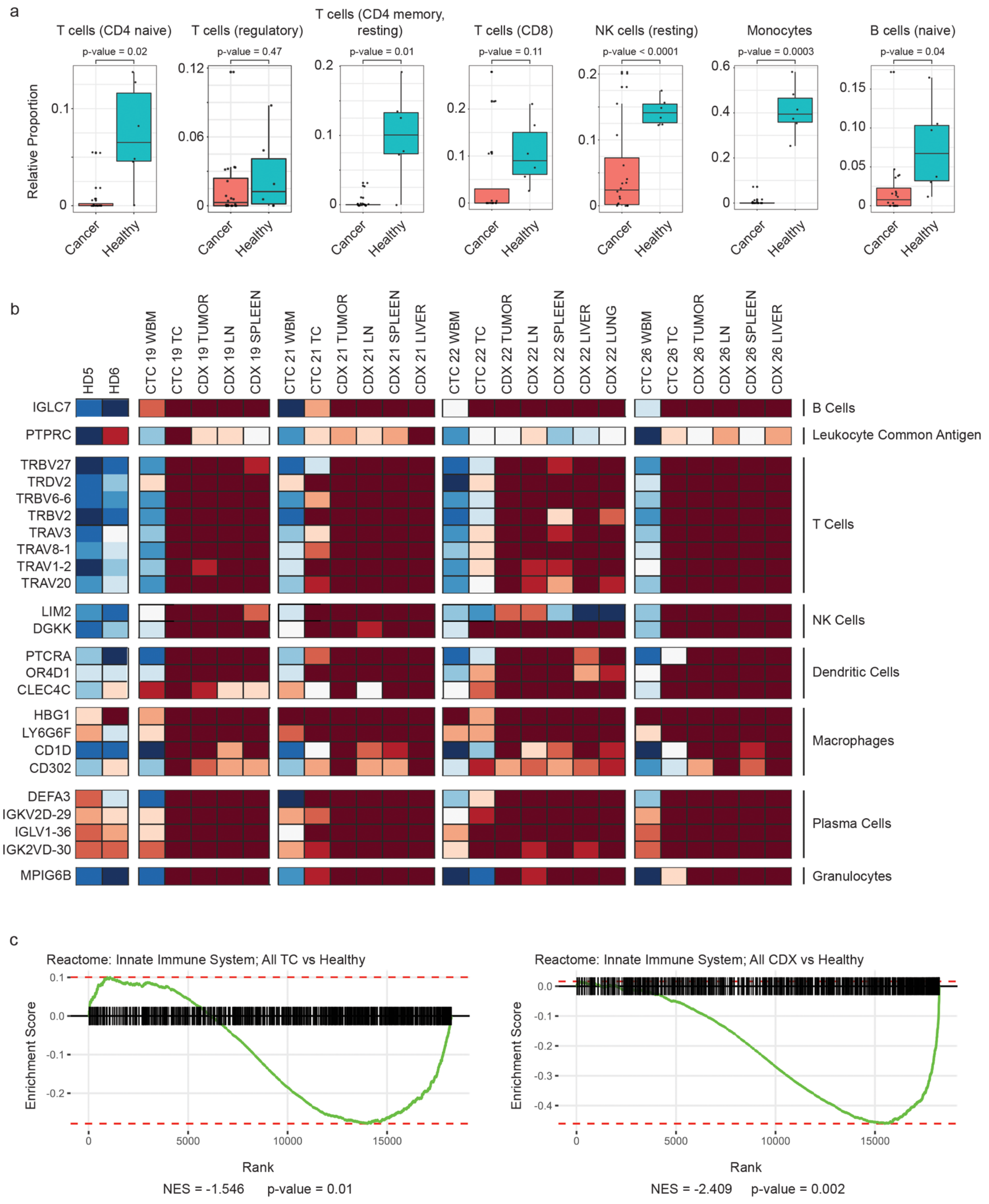

Figure S2. Immune cells depleted from TC and CDX compared to WBM and HDs. (a) Box plots of the relative proportion of immune cells signatures from CIBERSORT deconvolution indicates a depletion of T and B cells, as well as natural killer (NK) cells and monocytes. (b) Panels of genes that are highly enriched in single-cell immune signatures from Protein Atlas were extracted from the RNA-sequencing, confirming a depletion of immune cells within cancer samples except for corresponding patient whole blood. (c) Reactome pathway analysis was performed in comparisons between CTC culture (TC) or CDX samples against healthy. Both analyses indicate a depletion of innate immune cell system genes in either sample type. Cancer: TC and CDX tumor; healthy: WBM and HDs.

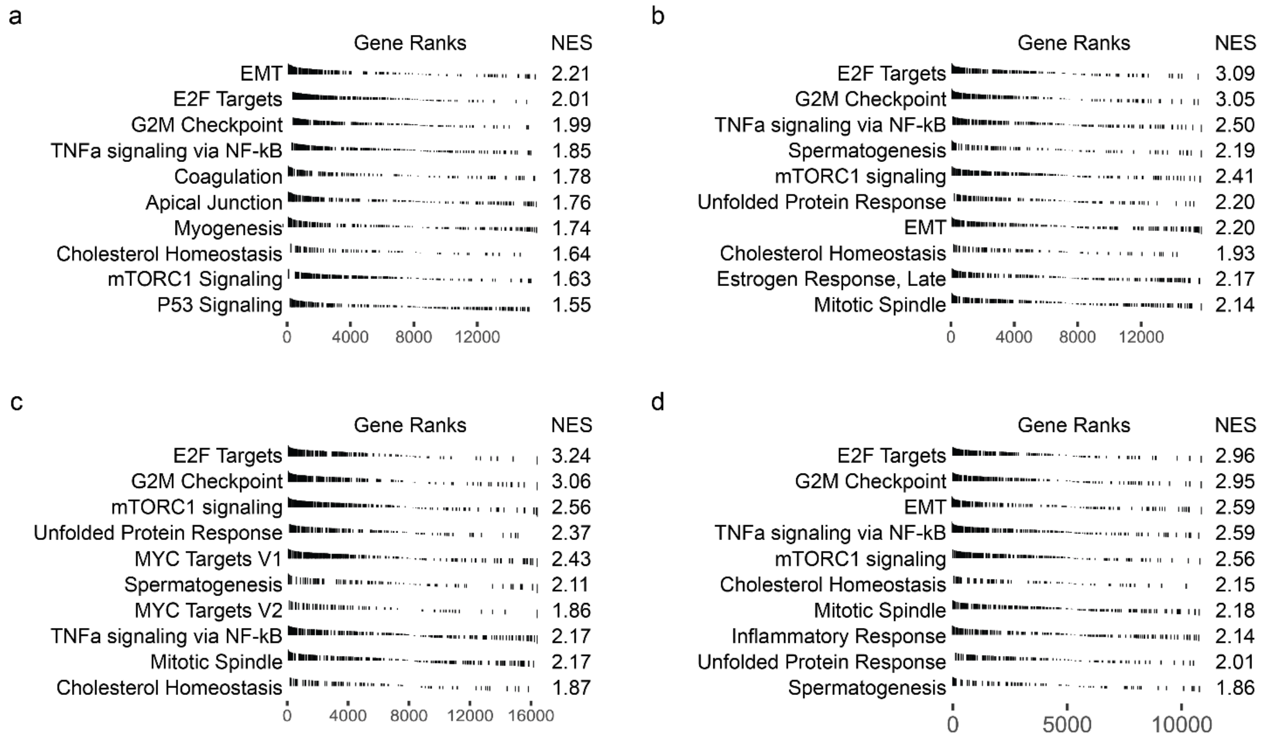

Figure S3. Cancer-related gene-sets are enriched in TC and CDXs. GSEA analysis was performed to evaluate enrichment in all CTC-derived samples relative to whole blood samples in (a) patient #19, (b) patient #21, (c) patient #22, and (d) patient #26. TC: cultured CTCs.

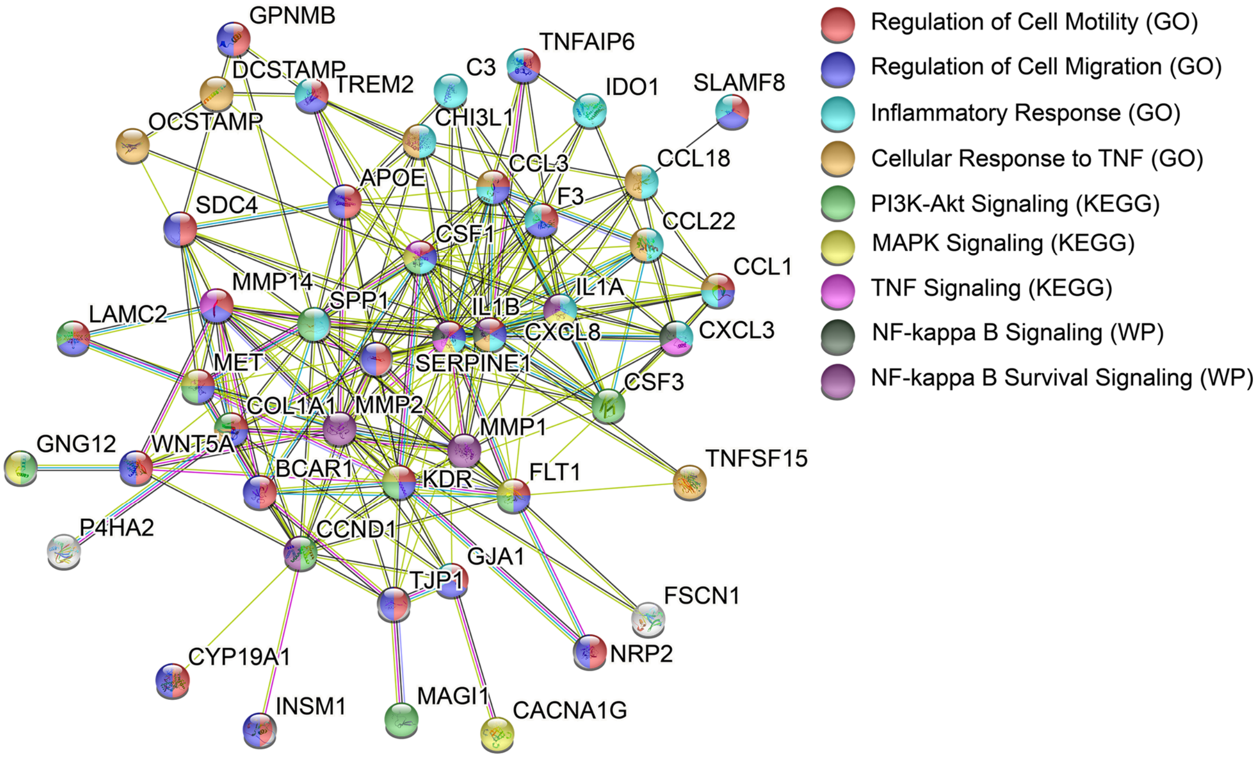

Figure S4. Functional network analysis reveals potential interactome circuitry unique to CTC cultures. A functional network was generated through input of the 199 gene prospective CTC culture signature in Table S3 into String-DB, using the whole human genome as background. Nodes represent all proteins produced by a single, protein-coding gene locus, and are colored by associated gene ontology (GO), KEGG pathway, or WikiPathway (WP) terms. White nodes represent proteins in a second shell of interactors. Edges or connecting lines represent protein-protein interactions. Known interactions are depicted as teal (from curated databases) or magenta (experimentally determined) edges. Predicted interactions are depicted as green (gene neighborhood), red (gene fusions), blue (gene co-occurrence), yellow (from text mining), black (co-expression), or violet (protein homology). Disconnected nodes were not plotted. All enriched terms showed a FDR < 0.05.

Table S1 – Differential gene analysis summarizing various cancer-agnostic and patient-specific comparisons.

Sample	Control	Upregulated Genes	Downregulated Genes
All CTC-derived samples	Whole blood samples	848	21
Cultured CTCs	Whole blood samples	1828	1165
CDX Tissues	Whole blood samples	3129	3419
Cultured CTCs	CDX Tissues	1836	2731
**CTC 19 Specific Analysis**			
All CTC-derived samples	Whole blood samples	213	12
Cultured CTCs	Whole blood samples	290	9463
CDX Tissues	Whole blood samples	2586	3122
Cultured CTCs	CDX Tissues	9279	191
**CTC 21 Specific Analysis**			
All CTC-derived samples	Whole blood samples	688	187
Cultured CTCs	Whole blood samples	474	656
CDX Tissues	Whole blood samples	1604	2685
Cultured CTCs	CDX Tissues	244	626
**CTC 22 Specific Analysis**			
All CTC-derived samples	Whole blood samples	796	256
Cultured CTCs	Whole blood samples	1533	791
CDX Tissues	Whole blood samples	1970	3531
Cultured CTCs	CDX Tissues	733	2602
**CTC 26 Specific Analysis**			

All CTC-derived samples	Whole blood samples	359	65
Cultured CTCs	Whole blood samples	1138	1297
CDX Tissues	Whole blood samples	1570	3011
Cultured CTCs	CDX Tissues	1323	2322

Table S2 – Top 10 most up- and down-regulated genes, patient-specific.

Upregulated (Gene, log2-fold change)			
**CTC 19**	**CTC 21**	**CTC 22**	**CTC 26**
GDA (25.8)	FOXA2 (24.5)	GJB1 (22.9)	NR2F2 (25.7)
IVL (25.7)	DSC3 (24.4)	NES (22.6)	CCN1 (25.0)
CCL17 (25.6)	MT1G (24.3)	FGA (21.8)	CCDC74A (13.4)
SYNPO2L (24.7)	IVL (23.6)	FLRT2 (21.2)	CERNA2 (12.2)
FRMPD2 (24.4)	HOXD9 (23.5)	GAL (20.7)	CXCL13 (11.7)
KCNA1 (24.3)	MT1M (23.4)	COL3A1 (20.0)	UCHL1 (11.5)
SIX3 (23.7)	GJB1 (23.1)	CHRD (19.9)	HMX2 (11.1)
GRIN2A (23.1)	PDPN (23.06)	S100A16 (19.9)	INHBA (11.0)
CCDC74A (13.0)	RASD2 (22.6)	OVOL1 (19.7)	VSTM2L (10.9)
BCAR1 (12.6)	FLRT2 (22.6)	CALB2 (19.7)	EFNB2 (10.7)
**Downregulated (Gene, log2-fold change)**			
XIST (-28.2)	XIST (-28.5)	RPS4Y1 (-30.0)	RPS4Y1 (-30)
CLEC4OP (-7.9)	SIGLEC14 (-9.4)	EIF1AY (-27.0)	EIF1AY (-26.4)
IGHV7-4-1 (-7.9)	ZFP57 (-7.3)	TXLNGY (-26.6)	TXLNGY (-25.8)
HDC (-7.8)	NRGN (-6.4)	KDM5D (-26.2)	UTY (-25.7)
SPATC1L (-7.7)	GZMH (-6.2)	DDX3Y (-25.6)	HSPA7 (-9.2)
IGLC5 (-6.8)	GZMM (-6.1)	GSTM1 (-10.4)	ZFP57 (-8.2)
LYPD2 (-6.6)	PTCRA (-6.1)	UTY (-10.1)	GSTM1 (-8.0)
MS4A2 (-6.4)	TRDC (-6.1)	LYPD2 (-8.0)	LCN8 (-7.7)
VMO1 (-5.6)	TMEM40 (-5.9)	LCN8 -7.6)	KRT72 (-7.3)
FCER1A (-5.6)	ITGA2B (-5.9)	PRKY (-7.2)	RNASE3 (-7.1)

Table S3 – A prospective CTC culture signature based on enriched expression in CTC cultures relative to patient-matched controls.

A4GALT	CP	HSD17B14	NRP2	SPR
ADAMDEC1	CRABP2	HSPB7	OCSTAMP	SPRY4
ADRA2B	CSF1	IDO1	OVOL1	SRRM3
AGRN	CSF3	IGSF3	P4HA2	SSTR2
AHRR	CSPG4	IL1A	PAQR5	ST18
AK4	CTSK	IL1B	PCARE	STC2
ANKRD1	CXCL3	IL4I1	PCGF2	STEAP1
AOC1	CXCL8	INHBA-AS1	PDCD1LG2	STRA6
AOX1	CYP19A1	INSM1	PDLIM4	SVEP1
APOC1	CYP27B1	KDR	PHF24	SYT12
APOE	DCSTAMP	KLK4	PIR	TCEAL9
ARMC9	DEPP1	KRT78	PLOD2	TFPI2
ASPHD1	DGKI	KRT79	PLTP	TGM2
ATF3	DIRAS2	LAMC2	PNCK	TIE1
BCAN-AS1	DPYSL3	LGMN	PNPLA3	TJP1
BCAR1	DUSP4	LINC00520	PPIC	TM4SF1
BEND3P2	EBI3	LINC01010	RAB42	TMEM132A
BHLHE41	EMP1	LINC01283	RAB7B	TMEM200B
C15orf48	EMX1	LINC01426	RAI14	TMEM45A
C1QTNF1	ENTHD1	LINC01827	RASAL2	TMEM51-AS1
C2CD4B	EPB41L1	LINC02099	RBP1	TNFAIP6
C3	EPOP	LINC02137	RGS16	TNFAIP8L3
C6orf223	F3	LINC02244	RGS20	TNFSF15
CA12	FAM124A	LIPG	RHBDF1	TREM2
CACNA1G	FJX1	LRRC32	RN7SL368P	TRIM6
CAVIN1	FLRT2	MAGI1	RNASE1	TSKU

CCDC74A	FLT1	MET	RPLP0P2	TTC23
CCL1	FSCN1	MMP1	RRAD	UST-AS2
CCL18	GAL	MMP14	RTN4RL2	VWA1
CCL22	GDA	MMP2	SCG5	WNT5A
CCL3	GDF15	MRC1	SDC2	ZMIZ1-AS1
CCM2L	GEM	MS4A6E	SDC4	
CCND1	GJA1	MT1G	SERPINE1	
CD109	GJA5	MT1H	SEZ6L2	
CD276	GJB2	MT1L	SHC4	
CD83	GNG12	MT1M	SLAMF8	
CHI3L1	GPC4	MUCL1	SLAMF9	
CHST3	GPNMB	MYPN	SLC1A2	
CLCNKA	GPX3	NCS1	SLC41A2	
CLLU1-AS1	GSDME	NECTIN4	SLC7A11	
COL1A1	HRH1	NES	SLCO2B1	
COL22A1	HSD11B1	NPR1	SPP1	

Table S4 – A prospective CDX signature based on enriched expression in CDX primary tumor samples.

ABCA12	CXCL9	KCNN1	PIEZO2	TFPI2
ADAMTS12	DMD	KIAA1549L	POU2F3	TSPAN11
BARX2	EMX1	LHX2	PRELID1P5	TSPAN12
BCAR1	ENOX1	LINC01055	RHOV	
CCL1	GJC1	LINC01258	RRAD	
CCL22	HNRNPA1P30	LINC02227	SLC12A8	
CCN1	IGSF3	LSAMP	SYNPO2L	
COL1A1	IL13	OSR2	TBXT	

Table S5 – TCGA pan-cancer atlas was queried to identify genes whose expression correlated with poorer overall survival.

Gene	Hazard Ratio	P-value
BCAR1	1.2 (1.0 - 1.5)	0.027
COL1A1	1.6 (1.3 - 2.0)	< 0.001
IGSF3	1.3 (1.1 - 1.6)	0.005
RRAD	1.4 (1.1 - 1.7)	0.002
TFPI2	1.5 (1.2 - 1.8)	< 0.001
CCL22	1.1 (0.91 - 1.4)	0.278
EMX1	0.98 (0.8 - 1.2)	0.807

Table S6 – Oncogenic mutations that were either FFPE, CTC culture, or CDX tumor-exclusive in Patient #19.

Gene	cDNA_change	Oncogenic	Source	TotReads	VariantAF
**KRAS**	chr12 c.35G>C	Yes	FFPE	144	0.194444
**KLF5**	chr13 c.81C>A	Yes	FFPE	14	0.142857
**E2F3**	chr6 c.1268T>G	Yes	FFPE	69	0.246377
**E2F3**	chr6 c.9G>A	Yes	FFPE	12	0.166667
**COP1**	chr1 c.1633C>T	Yes	Tumor	109	0.146789
**COP1**	chr1 c.1641C>T	Yes	Tumor	108	0.148148
**COP1**	chr1 c.1644A>T	Yes	Tumor	106	0.150943
**COP1**	chr1 c.582T>C	Yes	Tumor	73	0.616438
**COP1**	chr1 c.589A>G	Yes	Tumor	75	0.133333
**CDK8**	chr13 c.585T>A	Yes	Tumor	41	0.170732
**CDK8**	chr13 c.603C>T	Yes	Tumor	40	0.175
**CDK8**	chr13 c.606T>A	Yes	Tumor	40	0.175
**CDK8**	chr13 c.610C>T	Yes	Tumor	39	0.153846
**CDK8**	chr13 c.615T>C	Yes	Tumor	40	0.15
**CDK8**	chr13 c.618T>C	Yes	Tumor	39	0.153846
**CDK8**	chr13 c.624A>G	Yes	Tumor	40	0.15
**CDK8**	chr13 c.627G>A	Yes	Tumor	40	0.15
**MAP2K2**	chr19 c.660C>A	Yes	Tumor	21	0.333333
**GTF2I**	chr7 c.3105C>T	Yes	Tumor	100	0.33

Table S7 – Oncogenic mutations that were either FFPE, CTC culture, or CDX tumor-exclusive in Patient #21.

Gene	cDNA_change	Oncogenic	Source	TotReads	VariantAF
**NADK**	chr1 c.1212G>A	Yes	FFPE	47	0.489362
**BCL9**	chr1 c.1728G>A	Yes	FFPE	77	0.324675
**NTRK1**	chr1 c.1887C>T	Yes	FFPE	82	1
**JAK1**	chr1 c.2199A>G	Yes	FFPE	106	0.613208
**MEF2D**	chr1 c.282C>T	Yes	FFPE	35	0.571429
**NUF2**	chr1 c.558A>T	Yes	FFPE	85	0.764706
**IKBKE**	chr1 c.717G>A	Yes	FFPE	24	0.333333
**MEF2D**	chr1 c.756A>G	Yes	FFPE	75	0.6
**RET**	chr10 c.2071G>A	Yes	FFPE	80	0.3375
**RET**	chr10 c.2712C>G	Yes	FFPE	20	0.6
**MLLT10**	chr10 c.433A>G	Yes	FFPE	150	0.506667
**WT1**	chr11 c.231C>A	Yes	FFPE	18	0.555556
**LRP5**	chr11 c.4431C>T	Yes	FFPE	26	0.576923
**FGF4**	chr11 c.616C>A	Yes	FFPE	18	0.611111
**HRAS**	chr11 c.81T>C	Yes	FFPE	73	1
**CCND2**	chr12 c.570C>G	Yes	FFPE	83	0.457831
**LRP6**	chr12 c.924T>C	Yes	FFPE	74	0.405405
**FLT1**	chr13 c.1145G>A	Yes	FFPE	229	0.248908
**TSHR**	chr14 c.154C>A	Yes	FFPE	133	0.639098
**HIF1A**	chr14 c.1744C>T	Yes	FFPE	179	0.597765
**NTRK3**	chr15 c.573C>T	Yes	FFPE	25	0.36
**CYP19A1**	chr15 c.790C>T	Yes	FFPE	114	0.45614
**PLCG2**	chr16 c.174T>C	Yes	FFPE	29	0.931034
**PLCG2**	chr16 c.731A>G	Yes	FFPE	74	0.851351
**STAT5B**	chr17 c.1101C>A	Yes	FFPE	136	0.705882

**STAT5A**	chr17 c.1902C>T	Yes	FFPE	73	0.273973
**ERBB2**	chr17 c.1963A>G	Yes	FFPE	37	0.810811
**STAT5A**	chr17 c.613C>A	Yes	FFPE	13	0.153846
**STAT5A**	chr17 c.616C>A	Yes	FFPE	13	0.153846
**TYK2**	chr19 c.2051T>G	Yes	FFPE	46	0.391304
**RRAS**	chr19 c.333C>T	Yes	FFPE	81	1
**ARHGAP35**	chr19 c.3492G>T	Yes	FFPE	129	0.852713
**DOT1L**	chr19 c.4252G>C	Yes	FFPE	93	0.44086
**DOT1L**	chr19 c.4327G>T	Yes	FFPE	38	0.552632
**INSR**	chr19 c.783C>T	Yes	FFPE	63	0.444444
**XPO1**	chr2 c.1875T>G	Yes	FFPE	81	0.530864
**IRS1**	chr2 c.2004C>A	Yes	FFPE	129	0.674419
**IRS1**	chr2 c.2412A>G	Yes	FFPE	79	0.607595
**ALK**	chr2 c.2535T>C	Yes	FFPE	105	1
**ALK**	chr2 c.4472A>G	Yes	FFPE	138	0.630435
**ALK**	chr2 c.4587C>G	Yes	FFPE	116	0.698276
**PAK5**	chr20 c.1532G>A	Yes	FFPE	91	0.285714
**NCOA3**	chr20 c.1758G>C	Yes	FFPE	130	0.984615
**PLCG1**	chr20 c.2438T>C	Yes	FFPE	56	0.571429
**AURKA**	chr20 c.91T>A	Yes	FFPE	141	1
**BCL6**	chr3 c.1161C>T	Yes	FFPE	88	1
**GATA2**	chr3 c.15C>G	Yes	FFPE	9	1
**MECOM**	chr3 c.3036G>A	Yes	FFPE	161	0.391304
**MAP3K13**	chr3 c.333C>T	Yes	FFPE	150	0.566667
**ATXN7**	chr3 c.791A>G	Yes	FFPE	80	0.5375
**MST1R**	chr3 c.965G>A	Yes	FFPE	100	0.29
**KDR**	chr4 c.1416A>T	Yes	FFPE	26	0.384615
**KIT**	chr4 c.1621A>C	Yes	FFPE	158	0.487342

**KIT**	chr4 c.2586G>C	Yes	FFPE	75	0.413333
**SFRP2**	chr4 c.772C>G	Yes	FFPE	193	0.419689
**TRIP13**	chr5 c.1105T>C	Yes	FFPE	172	0.715116
**ARHGEF28**	chr5 c.1631C>T	Yes	FFPE	31	0.870968
**ARHGEF28**	chr5 c.1680A>G	Yes	FFPE	69	0.710145
**ARHGEF28**	chr5 c.1754G>A	Yes	FFPE	53	0.660377
**ARHGEF28**	chr5 c.2283C>T	Yes	FFPE	43	0.837209
**ARHGEF28**	chr5 c.2338C>A	Yes	FFPE	37	0.810811
**FLT4**	chr5 c.3971G>T	Yes	FFPE	60	0.9
**FLT4**	chr5 c.445A>G	Yes	FFPE	71	0.929577
**ARHGEF28**	chr5 c.673T>C	Yes	FFPE	52	0.75
**ARHGEF28**	chr5 c.851C>A	Yes	FFPE	32	0.78125
**ARHGEF28**	chr5 c.945T>C	Yes	FFPE	26	0.846154
**SGK1**	chr6 c.1005C>T	Yes	FFPE	58	0.896552
**JARID2**	chr6 c.1107C>T	Yes	FFPE	136	0.198529
**SGK1**	chr6 c.307A>T	Yes	FFPE	57	0.894737
**ROS1**	chr6 c.6637G>A	Yes	FFPE	44	0.204545
**ROS1**	chr6 c.6682A>C	Yes	FFPE	22	0.227273
**ROS1**	chr6 c.6686C>G	Yes	FFPE	22	0.227273
**DEK**	chr6 c.849G>A	Yes	FFPE	32	1
**DEK**	chr6 c.9C>T	Yes	FFPE	65	1
**CARD11**	chr7 c.1245C>T	Yes	FFPE	78	0.807692
**CARD11**	chr7 c.1599C>T	Yes	FFPE	34	0.823529
**MGAM**	chr7 c.2925C>A	Yes	FFPE	24	1
**GTF2I**	chr7 c.3105C>T	Yes	FFPE	111	0.279279
**MGAM**	chr7 c.3822T>C	Yes	FFPE	5	1
**MGAM**	chr7 c.3888T>C	Yes	FFPE	26	1
**MGAM**	chr7 c.4641T>C	Yes	FFPE	11	0.545455

**HGF**	chr7 c.910G>A	Yes	FFPE	101	0.287129
**NRG1**	chr8 c.1046T>C	Yes	FFPE	67	0.402985
**NRG1**	chr8 c.113G>A	Yes	FFPE	27	0.555556
**UBR5**	chr8 c.3957A>G	Yes	FFPE	218	0.376147
**UBR5**	chr8 c.7635A>C	Yes	FFPE	46	0.26087
**NR4A3**	chr9 c.1050G>A	Yes	FFPE	141	0.567376
**SYK**	chr9 c.1521C>T	Yes	FFPE	152	0.421053
**SYK**	chr9 c.1545T>C	Yes	FFPE	124	0.427419
**VAV2**	chr9 c.1780A>G	Yes	FFPE	100	0.38
**JAK2**	chr9 c.2490G>A	Yes	FFPE	46	1
**JAK2**	chr9 c.489C>T	Yes	FFPE	89	0.539326
**NR4A3**	chr9 c.590C>A	Yes	FFPE	12	0.25
**EZHIP**	chrX c.573G>T	Yes	FFPE	79	0.405063
**AR**	chrX c.639G>A	Yes	FFPE	150	0.553333
**COP1**	chr1 c.133G>T	Yes	Tumor	5	0.4
**COP1**	chr1 c.1633C>T	Yes	Tumor	91	0.208791
**COP1**	chr1 c.1641C>T	Yes	Tumor	91	0.208791
**COP1**	chr1 c.1644A>T	Yes	Tumor	92	0.206522
**COP1**	chr1 c.522C>T	Yes	Tumor	102	0.313725
**COP1**	chr1 c.549G>C	Yes	Tumor	101	0.336634
**COP1**	chr1 c.582T>C	Yes	Tumor	101	0.851485
**EIF4E**	chr4 c.249T>C	Yes	Tumor	14	0.142857
**NOTCH2**	chr1 c.15C>T	Yes	TC	21	1
**NT5C2**	chr10 c.1647C>T	Yes	TC	53	0.339623
**MLLT10**	chr10 c.195G>A	Yes	TC	253	0.588933
**PGR**	chr11 c.1031G>C	Yes	TC	120	0.558333
**RPS6KB2**	chr11 c.1259C>T	Yes	TC	36	1
**GAB2**	chr11 c.1290T>C	Yes	TC	86	0.569767

**APLNR**	chr11 c.135C>A	Yes	TC	170	1
**LMO1**	chr11 c.156C>G	Yes	TC	233	0.450644
**PGR**	chr11 c.1978G>T	Yes	TC	191	0.481675
**GAB2**	chr11 c.2004A>G	Yes	TC	64	0.40625
**PGR**	chr11 c.2310C>T	Yes	TC	50	0.44
**PGR**	chr11 c.2658A>G	Yes	TC	166	0.506024
**WT1**	chr11 c.345C>T	Yes	TC	20	1
**NUP98**	chr11 c.4284G>A	Yes	TC	97	0.494845
**RPS6KB2**	chr11 c.807C>T	Yes	TC	191	1
**FLI1**	chr11 c.942C>G	Yes	TC	251	0.498008
**KSR2**	chr12 c.1428G>A	Yes	TC	161	0.416149
**KDM5A**	chr12 c.2594T>C	Yes	TC	124	0.427419
**HDAC7**	chr12 c.2886G>A	Yes	TC	104	0.557692
**ATF1**	chr12 c.327C>T	Yes	TC	85	0.458824
**LRP6**	chr12 c.3810C>T	Yes	TC	147	0.442177
**KDM5A**	chr12 c.4149C>T	Yes	TC	89	0.438202
**RAB35**	chr12 c.486C>T	Yes	TC	25	0.44
**HDAC7**	chr12 c.741G>A	Yes	TC	95	0.442105
**IRS2**	chr13 c.2169C>T	Yes	TC	157	1
**IRS2**	chr13 c.2448T>C	Yes	TC	90	1
**IRS2**	chr13 c.3170G>A	Yes	TC	76	1
**HIF1A**	chr14 c.1253C>T	Yes	TC	75	0.373333
**FOXA1**	chr14 c.1343G>A	Yes	TC	158	0.601266
**TCL1B**	chr14 c.277G>A	Yes	TC	198	0.505051
**AKT1**	chr14 c.726G>A	Yes	TC	98	0.489796
**LTK**	chr15 c.125G>A	Yes	TC	60	0.483333
**USP8**	chr15 c.2215A>G	Yes	TC	41	0.463415
**CYP19A1**	chr15 c.240A>G	Yes	TC	103	1

**IGF1R**	chr15 c.3129G>A	Yes	TC	65	1
**LTK**	chr15 c.680C>T	Yes	TC	65	0.338462
**PLCG2**	chr16 c.1149C>T	Yes	TC	297	0.511785
**SETD1A**	chr16 c.1153C>T	Yes	TC	257	0.48249
**PLCG2**	chr16 c.297A>G	Yes	TC	76	0.526316
**PLCG2**	chr16 c.3093T>C	Yes	TC	188	1
**PLCG2**	chr16 c.802C>T	Yes	TC	203	0.44335
**RPTOR**	chr17 c.2010T>C	Yes	TC	85	0.517647
**RPTOR**	chr17 c.2094A>G	Yes	TC	75	0.4
**RPTOR**	chr17 c.2526C>T	Yes	TC	495	1
**MSI2**	chr17 c.321A>G	Yes	TC	178	0.488764
**RPTOR**	chr17 c.3231C>T	Yes	TC	87	0.574713
**RPTOR**	chr17 c.90T>C	Yes	TC	38	0.421053
**SETBP1**	chr18 c.3388C>A	Yes	TC	211	0.464455
**SERPINB3**	chr18 c.805T>A	Yes	TC	259	0.610039
**DNMT1**	chr19 c.1389A>G	Yes	TC	167	0.449102
**RRAS**	chr19 c.397G>A	Yes	TC	326	0.53681
**MAP2K2**	chr19 c.453C>T	Yes	TC	258	0.527132
**JAK3**	chr19 c.631A>C	Yes	TC	210	0.542857
**MAP2K2**	chr19 c.660C>A	Yes	TC	15	1
**GNA11**	chr19 c.771C>T	Yes	TC	134	1
**PLCG1**	chr20 c.1851G>A	Yes	TC	353	0.475921
**ICOSLG**	chr21 c.1195G>A	Yes	TC	50	1
**XBP1**	chr22 c.19G>A	Yes	TC	35	0.342857
**PDGFRB**	chr5 c.2601A>G	Yes	TC	207	0.429952
**FGFR4**	chr5 c.28G>A	Yes	TC	75	0.48
**PDGFRB**	chr5 c.3252A>G	Yes	TC	43	0.44186
**NSD1**	chr5 c.6903G>C	Yes	TC	172	0.430233

**FGFR4**	chr5 c.702C>T	Yes	TC	167	1
**DDX4**	chr5 c.859A>G	Yes	TC	271	0.472325
**NOTCH4**	chr6 c.1044C>G	Yes	TC	206	0.446602
**ROS1**	chr6 c.1986G>A	Yes	TC	93	0.548387
**NOTCH4**	chr6 c.2504G>T	Yes	TC	108	0.453704
**NOTCH4**	chr6 c.255C>T	Yes	TC	72	0.527778
**NOTCH4**	chr6 c.2967A>C	Yes	TC	215	1
**ROS1**	chr6 c.303A>T	Yes	TC	134	0.544776
**H1-4**	chr6 c.455A>G	Yes	TC	36	0.5
**NOTCH4**	chr6 c.522A>G	Yes	TC	294	0.517007
**ROS1**	chr6 c.6247A>C	Yes	TC	30	0.266667
**NOTCH4**	chr6 c.813A>G	Yes	TC	184	0.407609
**NOTCH4**	chr6 c.815A>G	Yes	TC	184	0.407609
**NOTCH4**	chr6 c.852G>A	Yes	TC	200	0.39
**NOTCH4**	chr6 c.958A>G	Yes	TC	122	0.467213
**ESR1**	chr6 c.975G>C	Yes	TC	200	0.385
**EPHA7**	chr6 c.981G>A	Yes	TC	119	0.386555
**SMO**	chr7 c.1137G>A	Yes	TC	264	0.458333
**EGFR**	chr7 c.1509C>T	Yes	TC	63	0.380952
**EGFR**	chr7 c.1562G>A	Yes	TC	119	0.512605
**EGFR**	chr7 c.1887T>A	Yes	TC	154	0.409091
**PIK3CG**	chr7 c.2025C>T	Yes	TC	263	0.353612
**CARD11**	chr7 c.3276A>G	Yes	TC	112	0.508929
**MGAM**	chr7 c.3315T>C	Yes	TC	149	0.442953
**MGAM**	chr7 c.3969T>C	Yes	TC	185	0.486486
**MGAM**	chr7 c.5408T>C	Yes	TC	143	0.496503
**MGAM**	chr7 c.6389G>C	Yes	TC	206	1
**MGAM**	chr7 c.7272A>G	Yes	TC	145	0.524138

**SYK**	chr9 c.129G>A	Yes	TC	384	0.479167
**ABL1**	chr9 c.3324A>G	Yes	TC	175	1
**NR4A3**	chr9 c.718A>G	Yes	TC	36	0.277778
**XIAP**	chrX c.1268A>C	Yes	TC	26	1
**MED12**	chrX c.3930A>C	Yes	TC	58	1
**CRLF2**	chrX c.730G>A	Yes	TC	46	0.652174

Table S8 - Oncogenic mutations that were either FFPE, CTC culture, or CDX tumor-exclusive in Patient #22.

Gene	cDNA_change	Oncogenic	Source	TotReads	VariantAF
**FLT1**	chr13 c.1145G>A	Yes	FFPE	229	0.248908
**STAT5A**	chr17 c.613C>A	Yes	FFPE	13	0.153846
**STAT5A**	chr17 c.616C>A	Yes	FFPE	13	0.153846
**JARID2**	chr6 c.1107C>T	Yes	FFPE	136	0.198529
**MGAM**	chr7 c.3822T>C	Yes	FFPE	5	1
**NR4A3**	chr9 c.590C>A	Yes	FFPE	12	0.25
**COP1**	chr1 c.582T>C	Yes	Tumor	21	0.571429
**GNAS**	chr20 c.636G>A	Yes	Tumor	8	0.5
**NUF2**	chr1 c.686C>T	Yes	TC	22	0.409091

Table S9 - Oncogenic mutations that were either FFPE, CTC culture, or CDX tumor-exclusive in Patient #26.

Gene	cDNA_change	Oncogenic	Source	TotReads	VariantAF
**PIK3CD**	chr1 c.2292C>T	Yes	FFPE	6	0.333333
**GNB1**	chr1 c.908A>G	Yes	FFPE	2	1
**LRP6**	chr12 c.499G>A	Yes	FFPE	2	1
**SOX9**	chr17 c.1328C>T	Yes	FFPE	2	1
**HOXB13**	chr17 c.249T>C	Yes	FFPE	2	1
**YES1**	chr18 c.778C>A	Yes	FFPE	3	0.666667
**NOTCH3**	chr19 c.1736G>C	Yes	FFPE	3	0.666667
**NOTCH3**	chr19 c.1744C>A	Yes	FFPE	3	0.666667
**IRS1**	chr2 c.3073A>G	Yes	FFPE	3	0.666667
**NSD2**	chr4 c.2909G>A	Yes	FFPE	255	0.164706
**NOTCH4**	chr6 c.1794T>C	Yes	FFPE	2	1
**COP1**	chr1 c.1633C>T	Yes	Tumor	85	0.188235
**COP1**	chr1 c.1641C>T	Yes	Tumor	85	0.188235
**COP1**	chr1 c.1644A>T	Yes	Tumor	85	0.188235
**COP1**	chr1 c.1650A>G	Yes	Tumor	82	0.170732
**COP1**	chr1 c.428T>C	Yes	Tumor	15	0.2
**COP1**	chr1 c.432A>G	Yes	Tumor	15	0.333333
**COP1**	chr1 c.435A>G	Yes	Tumor	13	0.230769
**COP1**	chr1 c.438A>C	Yes	Tumor	15	0.133333
**COP1**	chr1 c.522C>T	Yes	Tumor	80	0.3125
**COP1**	chr1 c.549G>C	Yes	Tumor	81	0.308642
**COP1**	chr1 c.582T>C	Yes	Tumor	84	0.809524
**COP1**	chr1 c.591G>A	Yes	Tumor	88	0.25
**COP1**	chr1 c.615C>A	Yes	Tumor	50	0.36
**FGFR2**	chr10 c.1709G>T	Yes	Tumor	13	0.153846

**LMO2**	chr11 c.100G>T	Yes	Tumor	8	0.25
**IRS2**	chr13 c.2851G>A	Yes	Tumor	11	0.181818
**CDK8**	chr13 c.324C>T	Yes	Tumor	53	0.188679
**CDK8**	chr13 c.342T>A	Yes	Tumor	82	0.134146
**CDK8**	chr13 c.348A>C	Yes	Tumor	87	0.126437
**CDK8**	chr13 c.585T>A	Yes	Tumor	29	0.310345
**CDK8**	chr13 c.603C>T	Yes	Tumor	26	0.269231
**CDK8**	chr13 c.606T>A	Yes	Tumor	26	0.269231
**CDK8**	chr13 c.610C>T	Yes	Tumor	26	0.269231
**CDK8**	chr13 c.615T>C	Yes	Tumor	26	0.269231
**CDK8**	chr13 c.618T>C	Yes	Tumor	26	0.269231
**CDK8**	chr13 c.624A>G	Yes	Tumor	26	0.269231
**CDK8**	chr13 c.627G>A	Yes	Tumor	26	0.269231
**FOXA1**	chr14 c.878G>T	Yes	Tumor	10	0.2
**GNA13**	chr17 c.664G>C	Yes	Tumor	139	0.438849
**AKT2**	chr19 c.291G>A	Yes	Tumor	69	0.173913
**AKT2**	chr19 c.297G>A	Yes	Tumor	70	0.171429
**AKT2**	chr19 c.306C>T	Yes	Tumor	73	0.164384
**AKT2**	chr19 c.327C>T	Yes	Tumor	82	0.146341
**AKT2**	chr19 c.330C>G	Yes	Tumor	83	0.144578
**VAV1**	chr19 c.753A>G	Yes	Tumor	16	0.125
**SOS1**	chr2 c.653A>G	Yes	Tumor	11	0.181818
**GNAS**	chr20 c.125G>A	Yes	Tumor	10	0.2
**NCOA3**	chr20 c.90C>T	Yes	Tumor	13	0.153846
**FOXL2**	chr3 c.839C>T	Yes	Tumor	15	0.133333
**UBR5**	chr8 c.6714A>G	Yes	Tumor	11	0.181818
**MED12**	chrX c.5C>T	Yes	Tumor	9	0.222222
**HDAC7**	chr12 c.1620C>T	Yes	TC	20	0.55

**CD276**	chr15 c.471A>C	Yes	TC	121	0.512397
**CD276**	chr15 c.479C>T	Yes	TC	118	0.5
**SERPINB3**	chr18 c.836G>C	Yes	TC	396	0.997475
**INSR**	chr19 c.5C>G	Yes	TC	5	1
**ICOSLG**	chr21 c.1057T>C	Yes	TC	68	1
**DDX4**	chr5 c.993C>T	Yes	TC	315	0.492063
**H1-4**	chr6 c.455A>G	Yes	TC	108	0.490741
**MGAM**	chr7 c.2742A>C	Yes	TC	214	0.537383
**MGAM**	chr7 c.4820A>C	Yes	TC	40	0.2
**MGAM**	chr7 c.6389G>C	Yes	TC	273	0.52381

Table S10 – Table of cancer-associated genes with mutations that were exclusive to their respective organs in the CDX models. LN = Lymph node

**Liver Only**		**LN Only**	**Spleen Only**
MTOR	PIK3CG	KDR	COP1
DDR2	MYC	RUNX1T1	RHOA
SETDB1	NTRK2	RPS6KB2	FOXL2
RRAGC	SYK	NOTCH3	EIF4A2
MPL	ABL1	EPOR	MGAM
JUN	GATA3		LMO2
JAK1	SHOC2		YAP1
NOTCH2	FGFR2		HDAC7
MCL1	RET		IRS2
PAX8	NUP98		YY1
XPO1	FGF3		CD276
IRS1	RPS6KA4		MAPK3
CXCR4	FLI1		AURKB
PPARG	LRP5		STAT5B
RAF1	CCND2		SOX9
MST1R	NFE2		AURKA
TP63	ATF1		BCR
MITF	MDM2		
FOXP1	MSI1		
GATA2	HIF1A		
PIK3CA	SETD1A		
DCUN1D1	PPM1D		
GAB1	ALOX12B		
PDGFRB	STAT5A		

TLX3	MAP3K14		
H1-4	DNMT1		
ROS1	INSR		
H2AC17	ERG		
CARD11	MED12		
ETV1	BTK		

Table S11: PCR Primers used in this study.

Gene	Forward Primer (5'-3')	Reverse Primer (5'-3')
**VIM**	CCTGGATTTCCTCTTCGTGG	TCCGGGAGAAATTGCAGGAG
**GAPDH**	AATCCCATCACCATCTTCCAG	GTTGTCATACTTCTCATGGTTCAC
**CK18**	CAGAGACTGGAGCCATTACTTC	GCCTTTTACTTCCTCTTCGTGG
